# Genome-wide association analyses of genetic, phenotypic, and environmental risks in the age-related eye disease study

**Published:** 2010-12-17

**Authors:** Euijung Ryu, Brooke L. Fridley, Nirubol Tosakulwong, Kent R. Bailey, Albert O. Edwards

**Affiliations:** 1 Department of Health Sciences Research, Mayo Clinic, Rochester, MN; 2 Institute for Molecular Biology, University of Oregon, Eugene, OR

## Abstract

**Purpose:**

To present genome-wide association analyses of genotypic and environmental risks on age-related macular degeneration (AMD) using 593 subjects from the age-related eye disease study (AREDS), after adjusting for population stratification and including questionable controls.

**Methods:**

Single nucleotide polymorphism (SNP) associations with AMD for the non-Hispanic white population were investigated using a log-additive model after adjusting for population stratification. Replication of possible SNP-disease association was performed by genotyping an independent group of 444 AMD case and 300 control subjects. Logistic regression models were used to assess interaction effects between smoking and SNPs associated with AMD. Independent genetic risk effects among the disease-associated SNPs were also investigated using multiple logistic regression models.

**Results:**

Population stratification was observed among the individuals having a self-reported race of non-Hispanic white. Risk allele frequencies at established AMD loci demonstrated that questionable control subjects were similar to control subjects in the AREDS, suggesting that they could be used as true controls in the analyses. Genetic loci (complement factor H [*CFH*], complement factor B [*CFB*], the age-related maculopathy susceptibility 2 locus containing the hypothetical gene [*LOC387715*]*/*the high-temperature requirement A-1 [*HTRA1*], and complement component 3 [*C3*]) that were already known to be associated with AMD were identified. An additional 26 novel SNPs potentially associated with AMD were identified, but none were definitely replicated in a second independent group of subjects. Smoking did not interact with known AMD loci, but was associated with late AMD. Statistically independent genetic signals were observed within the Pleckstrin homology domain-containing family A member 1 (PLEKHA1) region near *LOC387715/HTRA1* and within a haplotype spanning exon 19 of the *C3* gene.

**Conclusions:**

Population stratification among Caucasian subjects from the multicentered AREDS was observed, suggesting that it should be adjusted for in future studies. The AREDS questionable control subjects can be used as control subjects in the AREDS genome-wide association study (GWAS). Smoking was an independent risk factor for advanced AMD in the AREDS subjects. There continues to be evidence that the 10q26 (age-related maculopathy susceptibility 2 gene [*ARMS2*]) locus spanning *PLEKHA1-LOC387715-HTRA1* and the *C3* gene may contain multiple independent genetic risks contributing to AMD.

## Introduction

Age-related macular degeneration (AMD) is a progressive neurodegenerative disease that is the leading cause of vision loss in older individuals [1]. The more advanced forms of AMD are associated with atrophy of the retinal pigment epithelium (geographic atrophy) or abnormal angiogenesis (exudation) [2]. AMD is inherited as a complex disease with multiple genetic and environmental risks [3]. Environmental and lifestyle risk factors that may modify an individual’s risk of developing AMD include smoking, alcohol consumption, obesity, a lower level of physical activity, and diet [4]. The strongest identifiable risk factors for AMD continue to be age, family history, genetics, and smoking [5].

Currently, two biologic pathways contain genetic variants contributing to AMD risk. Genetic variations in multiple complement genes [6-12] and in a locus on chromosome 10q26 [13] are major risks for the development of AMD. Variants in other regions such as the human leukocyte antigen (*HLA*) locus, mitochondrial genome, interleukin 8 (*IL8*), apolipoprotein E (*ApoE*), and factor I (*FI*), may alter the risk of AMD, but additional studies are needed due to inconsistent or absent replication in independent study groups [14-17].

The genetic epidemiology of AMD is currently being investigated using genome-wide association studies (GWASs). The 593 subjects from the age-related eye disease study (AREDS) were genotyped using the Illumina Human-1 Bead-Chip panel containing 109,270 single nucleotide polymorphisms (SNPs). The data set was deposited into the National Center for Biotechnology Information database of Genotypes and Phenotypes (dbGaP). The AREDS subjects have been used for replication studies [11,18-20], meta-analyses [21], gentic epidemiology [22-24], and candidate gene selection by us and other investigators [12,21,25,26], but an analysis of the GWAS that includes strict quality control and population stratification has not yet been presented.

The AREDS was initiated in 1990 to learn more about the clinical course, prognosis, and risk factors of AMD and cataract. Over 4,700 people, ages 55 to 80 years, were enrolled in the AREDS cohort. These individuals were randomized for treatment with placebo, antioxidants, zinc, or antioxidants plus zinc, and were followed for a minimum of five years [27]. Results of the clinical trial suggested that people with a high risk of developing advanced AMD lowered their risk of AMD by 25% when they were treated with a high dose combination of vitamin C, vitamin E, beta-carotene, and zinc [27]. The AREDS GWAS is a valuable data set because of the extensive phenotypic and epidemiological variables collected during the study.

We present a comprehensive analysis of the AREDS GWAS to search for novel genetic loci for AMD, their interactions with smoking, and multiple independent genetic risks within AMD loci. In doing so, we have applied genotype quality-control filters and assessed genetic association using additive models accounting for population stratification. We have also compared the questionable control group (see Methods section) to the control group to determine if they have allele frequencies at AMD risk loci that are more similar to true controls or to cases. We attempted replication of all SNP effects that exceeded a significance level (p value) of less than 0.0001 in an independent case-control study of AMD, using logistic regression under an additive genetic model.

## Methods

### AREDS subjects and GWAS

We downloaded genotype data from the AREDS database (NIH-dbGaP), controlled through dbGaP accession number phs000001.v1.p1. Of the 593 subjects included in the AREDS genome-wide scan, 395 cases and 198 controls were successfully genotyped. The distribution of AMD subtypes, control subtypes, smoking exposure, and race are shown in Table 1. The cases were graded using standard clinical definitions of AMD [27]. The control category included subjects with AMD category 1 in both eyes at all visits. AMD category 1 was defined as any drusen present being less than 63 μm and the total area of drusen being less than the area of a 125 μm circle. The questionable control categories ranged from 1 to 4. The questionable control category 1 is for subjects who have AMD category 2 at only one visit except the last, and the questionable control category 4 is for those who have AMD category 2 (small to intermediate drusen) in one eye at the last vist, but AMD category 1 for both eyes at all other visits.

**Table 1:** Summary of age-related eye disease study (AREDS) subjects genotyped on the Human 1 Bead-Chip panel and the subjects used for replication.

	**AREDS subjects**		**Replication subjects**	
**Variable**	**Cases**	**Controls**	**Cases**	**Controls**
Number of AREDS subjects genotyped	395	198	444	300
Early AMD (%)	14 (3.5%)	-	202 (45.5%)	-
Geographic atrophy (%)	134 (34%)	-	67 (15.1%)	-
Exudation (%)	197 (49.9%)	-	175 (39.4%)	-
Both (%)	50 (12.6%)	-	-	-
Control (%)	-	171 (86.4%)	-	300 (100%)
Questionable control (%)	-	27 (13.6%)	-	-
Mean age (SD) in years	79.6 (4.98)	77.6 (4.8)	77.3 (9.3)	69.6 (8.3)
Male: Female ratio	0.73	1.04	0.56	0.88
**Smoking status**				
% Never smoked	41.5%	49.5%	44.1%	50.3%
% Previously smoked	47.9%	44.4%	46.0%	41.7%
% Currently smoke	10.6%	6.1%	5.4%	5.0%
% Smoking Unknown	0%	0%	4.5%	3.0%
**Race**				
% White, non-Hispanic	99.0%	0.9495	98.9%	99.3%
% White, Hispanic	0%	0	1.1%	0.3%
% White, Unknown	0%	0	0%	0.3%
% Black, non-Hispanic	0. 5%	5.0%	0%	0%
% Other	0.5%	0%	0%	0%

The proportions of age related macular degeneration (AMD) subtypes, gender, age, and race are shown.

### Replication

SNPs from the AREDS genome-wide scan were analyzed using logistic regression under an additive genetic model. Those with p values of less than 0.0001 from loci not known to contribute to AMD were considered for replication using an independent sample of AMD cases and controls. The replication subjects consisted of 744 individuals including 444 AMD cases and 300 controls without AMD. The use of these subjects was approved by the institutional review board of the Mayo Clinic, written informed consent was obtained from all subjects, and the study was performed in accordance with the Helsinki declaration. Diagnosis was determined by review of fundus photographs as previously described [6]. Briefly, all subjects diagnosed with AMD had large drusen or more advanced findings, and controls had five or fewer hard drusen without pigment changes or more advanced findings. SNPs for replication were genotyped using either Illumina or TaqMan assays (Applied Biosystem, Foster City, CA) as previously described [11,21,26]. A log-additive model using logistic regression was used for single SNP association analysis.

### Quality-control filters

Quality-control filters were applied to the SNPs from the AREDS GWAS before the assessment of association with AMD. SNPs with a call rate less than 95%, HWE p values based on controls <1×10^−7^, and SNPs with a minor allele frequency less than 1% were removed from further analyses. Among 593 subjects, three subjects were further excluded, as they were considered possible first-degree relatives to the remaining subjects, based on pairwise identities determined by state calculations. Ninety-five percent of the SNPs passed the quality-control filters, leaving 103,895 SNPs for analysis.

### Population stratification

Assessment of population stratification was performed using a principal components method [28] with the SNPs that passed our quality-control thresholds across the genome found on the 100K Illumina SNP panel. Principal component analysis was first completed using all cases and controls for all races (n=590), followed by an analysis including only subjects whose self-reported race was white, non-Hispanic (n=576). All further analyses were corrected for the first two principal components.

### Genome-wide analysis

Single SNP analyses were performed using R, PLINK, and SAS version 9.1 (SAS Institute Inc., Cary, NC). Log-additive models using logistic regression were performed to examine the association between single SNPs and AMD status, as this protected against finding positive associations due only to differences in the heterozygote genotype frequencies between the AMD cases and controls [26]. To visualize the results, quantile-quantile (Q-Q) plots were drawn to compare the distribution of observed p values to the expected distribution under the null hypothesis of no association. Plots of the level of significance of association with AMD status versus chromosomal position (Manhattan plots) were presented to show the significance of the SNPs across genomes.

### Correction for known SNPs

We wished to determine if any SNPs would be associated with AMD after adjusting for known loci. The process of choosing SNPs to exclude known loci was as follows: First, we calculated single SNP p values for all SNPs in each known locus (complement factor H [*CFH*], complement factor I [*CFI*], complement component 2/complement factor B [*C2/CFB*], age-related maculopathy susceptibility 2 [*ARMS2*], and complement component 3 [*C3*] loci) after adjusting for the first two principal components, and we found the most significant SNP for each locus. Second, we calculated the extent of linkage disequilibrium (using r^2^) between the most significant SNP in each locus and the rest of the SNPs in that locus to determine if there were any significant SNPs not in high linkage disequilibrium (r^2^ less than 0.2). Third, we performed logistic regression to see if such SNPs had any additional effect after conditioning on the most significant SNP for each locus. Seven SNPs were thus obtained, statistically accounting for the genetic risk contributed to AMD by the known loci as follows: the *CFH* locus, rs1853883; *CFI* locus, rs6848178; *C2/CFB* locus, rs541862; *ARMS2* locus, rs2014307 and rs4565845; and *C3* locus, rs2230199 and rs433594. The two SNPs from the *ARMS2* locus (and the *C3* locus) were selected to represent that locus, as they were in low linkage disequilibrium (r^2^<0.2), they statistically significantly contributed to AMD, and they each made an independent contribution to AMD conditioning on the other SNP. To understand the independent genetic effects within the *ARMS2* locus (and *C3* locus), haplotype analyses were performed for using the score test implemented in R package haplo.stats [29]. We then repeated the GWAS analysis, performing single SNP analyses with adjustments for these seven SNPs and the first two principal components.

### Smoking interaction

For assessment of the interaction effect of SNPs significantly associated with AMD and smoking status, a logistic regression model under a log-additive genetic model was used, in which each significant SNP and smoking status were included in the main effects. An interaction effect for SNP smoking was also included, after adjusting for the first two principal components. Smoking status was coded as 1 if the subject was a former smoker or current smoker at the beginning of the study, or was coded as 0 if the subject had never smoked. The distribution of smokers is shown in Table 1.

## Results

### Questionable controls had greater similarity to controls than to cases

**Figure 1:**
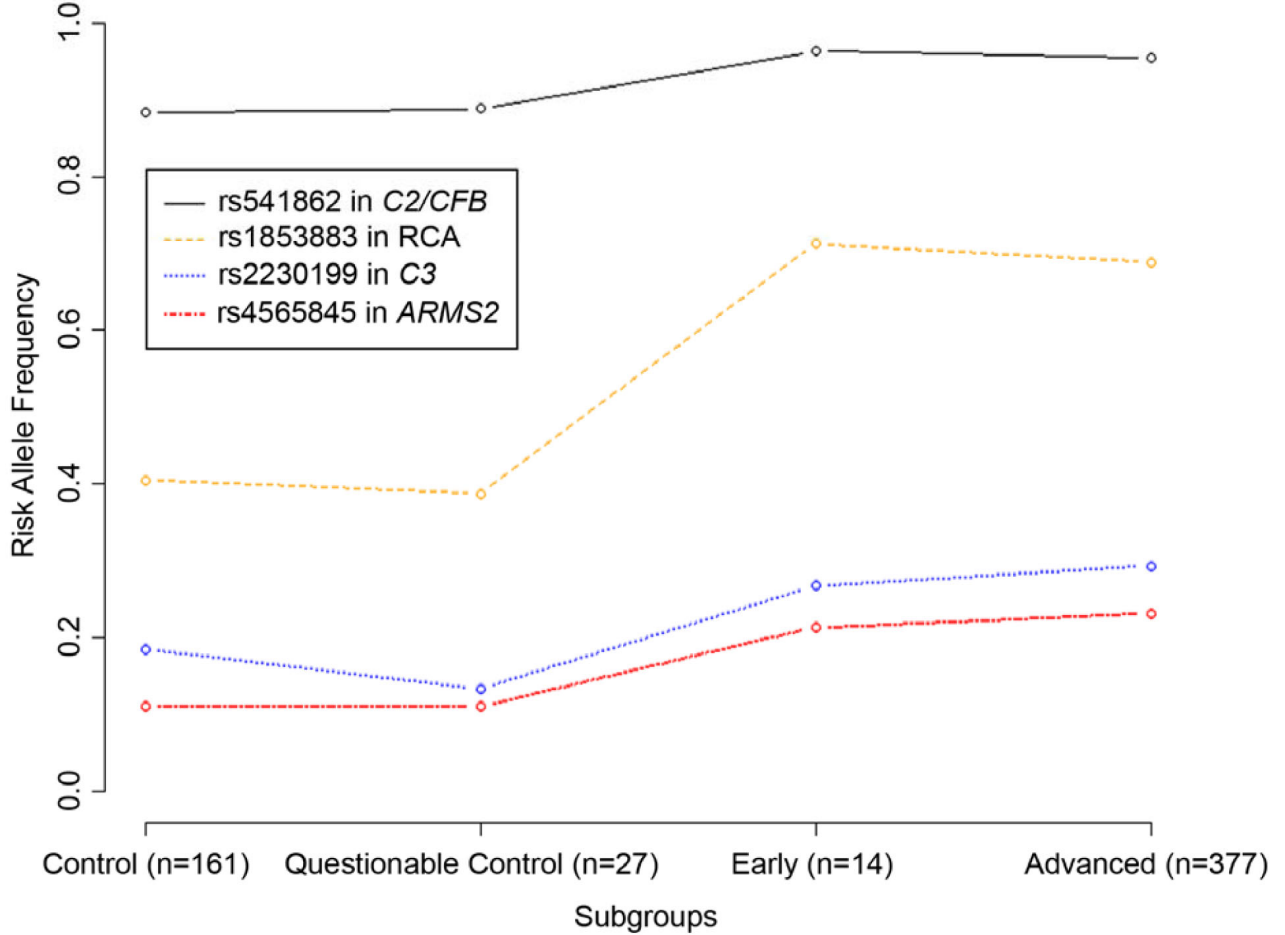
Comparison of the frequency of the risk allele for four single nucleotide polymorphisms (SNPs) known to contribute to age-related macular degeneration (AMD) in the age-related eye disease study (AREDS) “control” and “questionable control” subjects. Each of these SNPs has been consistently associated with AMD in multiple studies. The figure shows that the questionable control category was similar to the control category in terms of the frequency of the risk allele. Questionable control subjects can be used as controls in analyses using the AREDS data set. Abbreviations: *RCA* is the regulation of complement activation locus containing the gene encoding *CFH*; please see introduction for other gene and locus names.

The risk allele frequencies in the 27 questionable control subjects were compared to the other control and AMD subtypes using a two-sample test for equality of proportions (Figure 1). The risk allele frequencies in the questionable control group were very similar to those from the control group, but not from the cases. P values comparing questionable controls to control groups varied from 0.48 to 1, while those comparing them to cases varied from 1.04×10^−5^ to 0.06. Thus, the questionable controls could be combined with the controls for subsequent analyses.

### Population stratification

**Figure 2:**
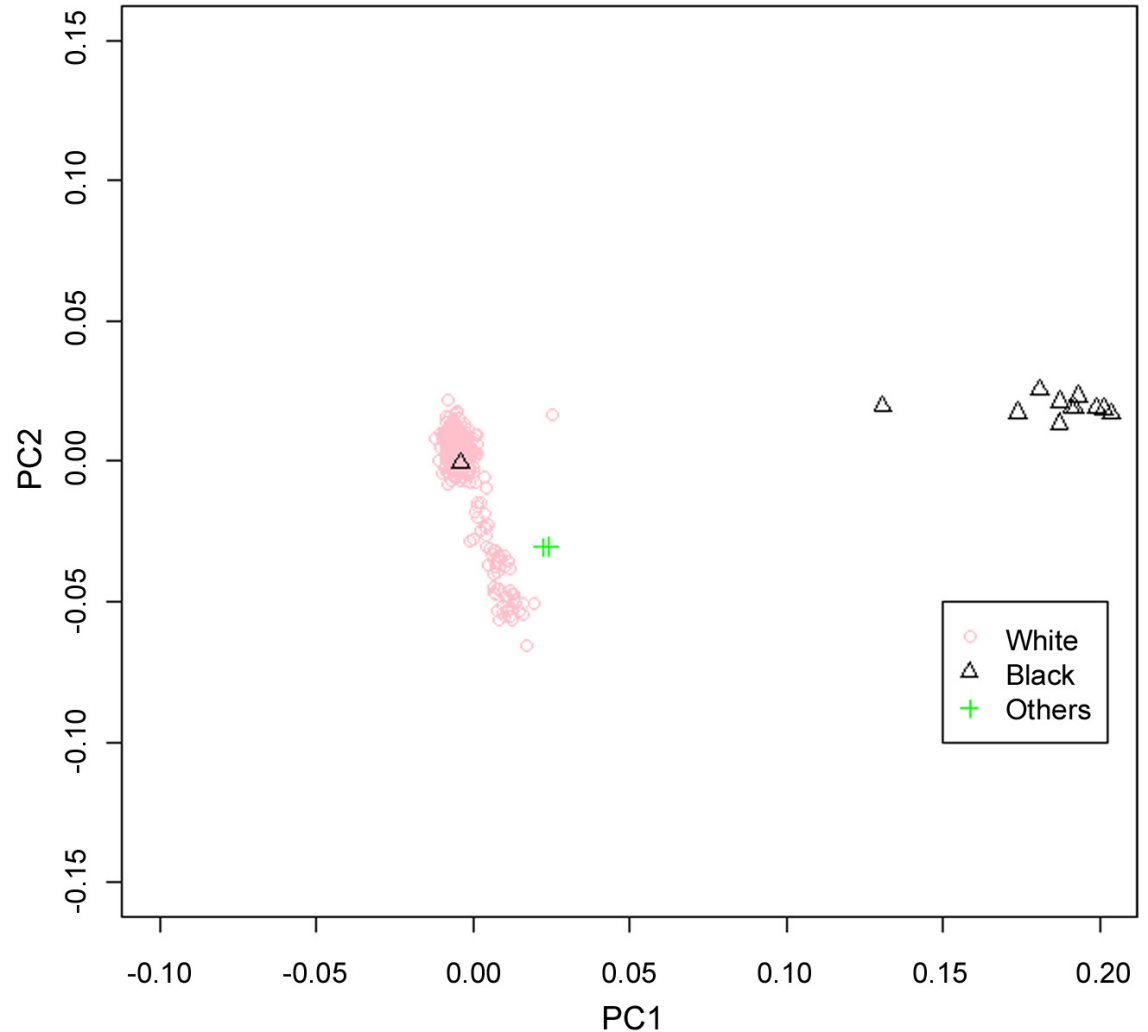
Population stratification in the age-related eye disease study (AREDS) subjects. Three race categories are presented in pink circle for white, non-Hispanics (White), in black triangle for blacks (Black), and in green cross for subjects who are neither white, non-Hispanics nor blacks. A plot of the first two principal components using 103,895 single nucleotide polymorphisms (SNPs) passing quality-control filters. Of the total 590 subjects, the self-reported race was white, non-Hispanic for 576, black for 12, and “other” for 2. The figure shows that one black subject and two other subjects were similar to the white subjects. There was also stratification among the white subjects, for which we corrected in subsequent analyses. The x-axis shows the first principal component axis (PC1), while the y-axis shows the second component (PC2).

To explore possible population stratification, a principal components approach was used to assess population stratification in all subjects and in those who self-reported their race as white, non-Hispanic (Table 1). Figure 2 shows a plot of the first two principal components, which discriminated blacks from non-Hispanic whites, with the exception of one self-defined black subject. The two individuals with unspecified race clustered close to the non-Hispanic whites. The figure shows that there was potential population stratification among the white, non-Hispanic subjects. For further analyses, we used subjects who self-reported as white, and corrected for population stratification among the white subjects.

### Genome-wide analysis

**Figure 3:**
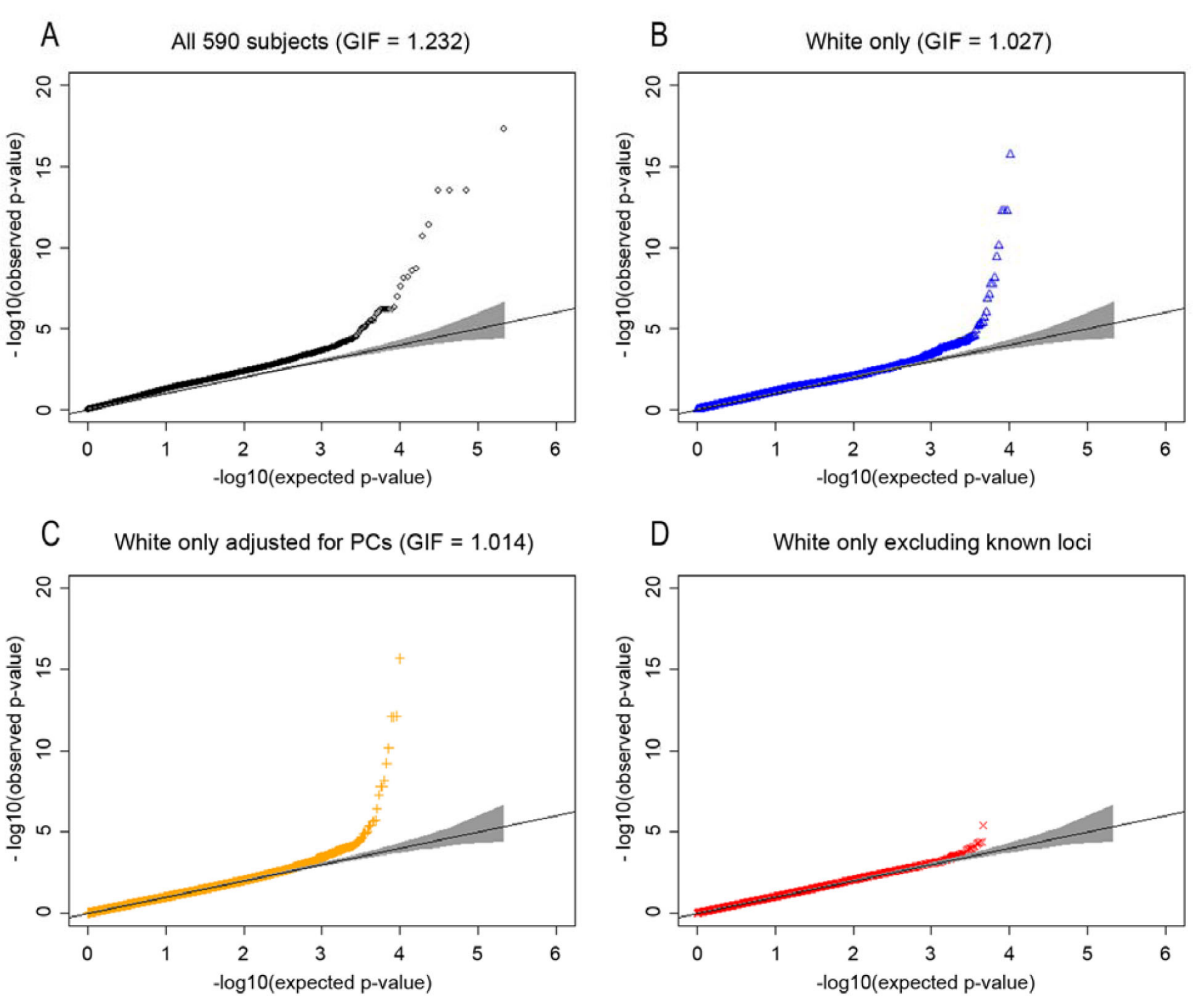
Quantile-quantile plots of the age-related eye disease study (AREDS) genome-wide association study (GWAS). **A**: Scatter plot of the −log10 (p values) expected under the null hypothesis of no genetic association versus the observed −log10 (p values) are shown for all subjects. **B**: This is a scatter plot as in **A**, but for white subjects only. **C**: This is a scatter plot of white subjects corrected for population stratification using principal components methods without exclusion of known loci. **D**: This is a scatter plot of white subjects corrected for population stratification using principal components methods with exclusion of known loci. **A**–**C**: These plots show that correction for population stratification reduced the genomic inflation factor from 1.23 to 1.01. **D**: This plot suggests that there is evidence for additional single nucleotide polymorphisms (SNPs) contributing to age-related macular degeneration (AMD) after statistically accounting for known loci.

**Figure 4:**
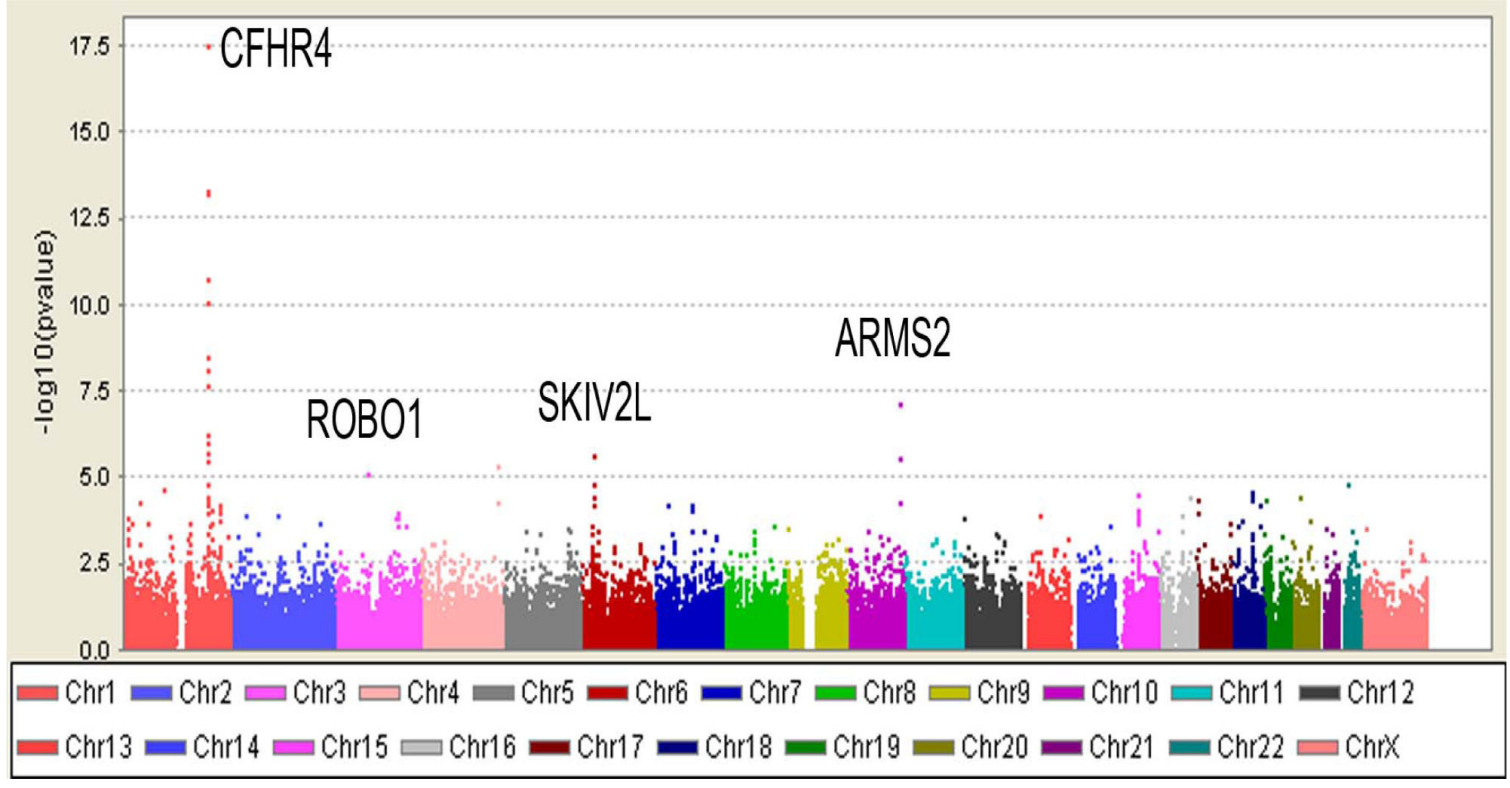
The association of single single nucleotide polymorphisms (SNPs) with age-related macular degeneration (AMD) across the genome. Significance across the genome is illustrated by plotting −log 10 (p values) from the log-additive genetic model corrected for population stratification. The loci with p values less than 0.00001 are labeled and the SNPs are provided in Table 2.

The association between each SNP and AMD risk was investigated under the log-additive genetic model. Figure 3 shows Q-Q plots of the association p values under four different scenarios. The Q-Q plot using all subjects (3A) clearly shows population stratification with an unacceptably high genomic inflation factor of 1.23. Using only white subjects and after adjusting for the first two principal components (Figure 3C), the genomic inflation factor was reduced to an acceptable level of 1.014. Figure 3D shows that there is evidence for additional SNPs associated with AMD after correction for SNPs already known to be associated with AMD. All seven SNPs were incorporated into a logistic regression model to correct for known association. Figure 4 shows the distribution of p values across the genome; individual SNPs with p values less than 0.00001 are labeled, and additional information is provided in Table 2.

**Table 2:** Replication of single nucleotide polymorphisms (SNPs) associated with age related macular degeneration (AMD) in the age-related eye disease study (AREDS) genome-wide association study (GWAS) and Replication subjects.

					**Minor allele frequency**		**Log-additive p-values**			
**Chromosome**	**SNP**	**Nearby gene**	**Base pair position (build 36.3)**	**Minor allele**	**Cases**	**Controls**	**HWE p-value**	**Adjusted for population stratification**	**Adjusted for population stratification and known loci†**	**Replication p-value**
1	rs11208590	RIMS3	40880736	C	0.3	0.41	0.23	5.49E-05	0.00096	0.49
1	rs2297634	ABCA4	94349556	C	0.52	0.38	0.66	2.27E-05	0.00422	0.71
1	rs1853883	CFHR4	1.95E+08	C	0.31	0.6	0.2	3.41E-18	-	*
1	rs2054780	YOD1	2.05E+08	T	0.15	0.07	0.91	9.69E-05	0.00387	0.94
1	rs287614	OBSCN	2.27E+08	A	0.51	0.38	0.29	8.59E-05	0.00393	0.24^1^
1	rs1150910	OBSCN	2.27E+08	A	0.51	0.38	0.29	7.33E-05	0.00341	0.24^1^
3	rs2055451 ^5^	ROBO1	79924819	T	0.25	0.37	0.2	9.34E-06	9.06E-05	0.11^5^
4	rs1447338	-	1.81E+08	G	0.22	0.35	0.51	5.02E-06	0.00587	0.17
4	rs13129209	-	1.81E+08	T	0.29	0.41	0.38	5.94E-05	0.03675	0.65
6	rs429608	SKIV2L	32038441	A	0.08	0.17	0.07	2.49E-06	0.03228	*
7	rs2267742	ADCYAP1R1	31107071	A	0.05	0.11	0.35	6.96E-05	0.00677	0.39
7	rs10275700	-	85428174	T	0.43	0.55	0.7	0.00007	0.00109	0.01^2^
7	rs10274362	-	85449561	G	0.52	0.4	0.48	9.73E-05	0.00191	0.01^2^
7	rs988426	-	85454805	T	0.42	0.55	0.96	7.43E-05	0.00121	0.01^2^
10	rs2014307	ARMS2	1.24E+08	T	0.27	0.43	0.45	7.48E-08	-	*
15	rs2713935	-	54333504	C	0.44	0.57	0.25	3.62E-05	0.00179	0.62^3^
15	rs6493856	TEX9	54474651	A	0.51	0.38	0.53	0.000098	0.0081	0.93^4^
16	rs8056814	CTRB1#	73809828	A	0.05	0.13	0.17	4.16E-05	0.0012	0.02
17	rs4268798	METT10D#	2313335	C	0.39	0.52	0.04	5.11E-05	0.0025	0.47
18	rs9950970 ^5^	DCC#	49117806	C	0.3	0.43	0.58	2.87E-05	4.81E-05	0.65
18	rs1367634 ^5^	DCC#	49118666	T	0.3	0.43	0.45	4.87E-05	7.41E-05	0.6
18	rs12954274 ^5^	DCC#	49119872	C	0.3	0.43	0.65	3.46E-05	5.44E-05	0.61
18	rs8086078	FBXO15	69950556	C	0.03	0.08	0.37	0.000072	0.00021	0.83
19	rs2230199	C3	6669387	C	0.29	0.18	0.3	4.89E-05	-	*
20	rs6137194 ^5^	-	20896861	C	0.38	0.25	0.7	4.35E-05	0.000046	0.8
22	rs9608466 ^5^	SEZ6L	24891478	A	0.04	0.11	0.84	1.68E-05	3.89E-06	0.34
4	rs6532378 ^5^	GRID2	93761791	A	0.33	0.34	0.64	0.0093	4.58E-05	0.28^6^
9	rs972925 ^5^	OR1Q1	1.24E+08	A	0.17	0.17	0.43	0.0043	7.44E-05	0.19
21	rs2838378 ^5^	-	44030783	C	0.42	0.44	0.41	0.0127	4.79E-05	0.06^7^

SNPs associated with AMD with a p-value of less than 0.0001 using the log-additive model with correction for population stratification are shown, except that only the most significant SNP in already established AMD risk loci are included. The last three rows show SNPs associated with AMD only after correction for known SNPs. The significance of attempted replication in the Replication subjects is shown in the last column. The association with AMD was not replicated for any of the SNPs. †The SNPs used for conditioning are listed in the methods section. *These SNPs are located within loci already known to be associated with AMD. No replication was attempted. Note that *CFHR4* is near *CFH* and *SKIV2L* is near *C2/CFB*. # Loci genotyped on Illumina platform (based on allele distribution p-values provided on the dbGaP website) and previously reported [26] ^1^ The p-value for rs435776 was used to represent rs287614 (r^2^=1) and rs1150910 (r^2^=0.96) ^2^The p-value for rs10245061 was used to represent rs10275700 (r^2^=0.89), rs10274362 (r^2^=1), and rs988426 (r^2^=0.89) ^3^ The p-value for rs2725854 was used to represent rs2713935 (r^2^=0.83) ^4^ The p-value for rs7170307 was used to represent rs6493856 (r^2^=0.86) ^5^ These SNPs were significantly associated with AMD after adjustment for known AMD SNPs and loci (please see Figure 3D) ^6^ The p-value for rs1349350 was used to represent rs6532378 (r^2^=1) ^7^ The p-value for rs1041456 was used to represent rs2838378 (r^2^=0.96).

### Association of individual SNPs with AMD

SNPs associated with AMD at a p value of less than 0.0001 before or after adjustment for SNPs already known to be associated with AMD are shown in Table 2. Note that we did not include any SNPs from known loci. There were 26 SNPs meeting these criteria before adjustment and nine SNPs after adjustment; six SNPs met both criteria. Replication in an independent group of subjects was attempted for all SNPs. Only one SNP reached nominal significance (*CTRB* locus) in the replication subject group, but the p value (0.02) would not be considered significant after Bonferroni correction for multiple testing. The value of incorporating population stratification and using the log-additive model is illustrated by the SNP (rs2230199) in *C3* in Table 2, which reached the statistical significance threshold (p<0.0001) only after correction of population stratification and using a log-additive genetic model (Fisher’s exact test; p=0.00048).

### Interaction between smoking and SNPs associated with AMD

Smoking was not associated with early AMD (OR=0.58, 95% CI=0.18–1.80. The p value equaled 0.34, but was associated with geographic atrophy (OR=1.62, 95% CI=1.07–2.44, p=0.02), exudative AMD (OR=1.51, 95% CI=1.00–2.26, p=0.05), and advanced AMD (OR=1.56, 95% CI=1.10–2.22, p=0.01) compared to control groups among the AREDS GWAS subjects. The replication sample showed similar results: early AMD (OR=0.87, 95% CI=0.60–1.25, p=0.45), geographic atrophy (OR=1.68, 95% CI=0.97–2.92, p=0.06), exudative AMD (OR=1.75, 95% CI=1.18–2.58, p=0.005), and advanced AMD (OR=1.73, 95% CI=1.22–2.46, p=0.002). All 59 SNPs associated with AMD at p values less than 0.0001 or already known to be associated were investigated for possible interactions with smoking status. Five SNPs (rs11208590, rs572515, rs7529589, rs12038333, and rs203674) reached nominal significance levels (with p values varying from 0.02 to 0.01), but none of the interactions would be considered significant after Bonferroni correction for multiple testing.

### Independent genetic effects within the *ARMS2* and *C3* loci

We observed statistically independent effects of both rs4565845 and rs2014307 across the *ARMS2* locus at chromosome 10q26 (see Correction for known SNPs in Methods section). SNP rs4565845 is located within the pleckstrin homology domain-containing family A member 1 (*PLEKHA1*) gene, while rs2014307 is located between *LOC387715* and the high-temperature requirement A-1 (*HTRA1*) promoter. We inspected the haplotypes formed by the six SNPs genotyped in the AREDS GWAS (rs4146894, rs4752692, rs4565845, rs7097701, rs2292623, and rs2014307) across 62 kb of the 10q26 locus (*PLEKHA1*-*LOC387715*-*HTRA1* promoter). The SNP most highly associated with AMD was rs2014307, which captures information from both risk and protective haplotypes across the *ARMS2* locus. Formation of haplotypes with and without rs4752692 did not appear to lead to any refinement of the association of haplotypes with AMD. Specifically, the addition of rs4752692 split the haplotype formed by the other five SNPs (GC-TAC) that were highly associated with AMD (p=7.4×10^−12^) into two haplotypes (GCCTAC, 3.1×10^−7^ and GCATAC, 1.0×10^−4^) that were both associated with AMD.

We also observed independently, statistically significant effects of two SNPs (rs433594 and rs2230199) across the *C3* locus. The non-synonymous SNP rs2230199 forms a high-risk haplotype with another non-synonymous SNP (rs1047286), and the effects of these two SNPs cannot be clearly distinguished [12]. SNP rs433594 is only modestly associated with AMD in the AREDS GWAS (p=0.01). However, four SNPs in this region near exon 19 (rs237554, rs423490, rs433594, and rs428453) form a haplotype (GGTC; with a frequency of 0.1 in cases and 0.18 in controls) that is associated with AMD (p=3×10^−4^) at a level similar to the non-synonymous SNP rs2230199 (3.4×10^−5^). The GGTC haplotype spanning exon 9 does not appear to be in linkage disequilibrium with the haplotype tagged by rs2230199 that spans exons 3–9.

## Discussion

A genome-wide scan performed on 593 subjects from the AREDS was released in the database of Genotypes and Phenotypes (dbGaP) without an associated publication describing the genetic features of the data set. The AREDS genome-wide scan data set has been used extensively for genetic studies on AMD, as detailed in the introduction. Because of the ongoing analyses using the AREDS subjects and the AREDS genome-wide scan data set, we felt that a detailed analysis of the genome-wide scan data set might provide valuable guidance on its use in AMD research.

The AREDS data set has been studied previously in two reports [20,26]. These studies did not analyze the data set using the actual genotypes from the AREDS data set. Instead, both studies obtained p values from the AREDS dbGaP website to select the SNPs for replication studies. Herein, we have performed a primary analysis of the AREDS data set using standard approaches, including log-additive models and correction for population stratification.

Prior to initiating the analysis of the genome-wide scan data set, we sought to determine the heterogeneity within the various categories of control subjects within the AREDS. The phenotypic range of drusen that has been allowed for control subjects in AMD studies has been controversial. Many groups use an extreme phenotype approach in which only a few hard drusen (without more advanced features such as pigment changes) are allowed in control subjects, based on fundus photography [6,30,31]. Other groups have used clinical examination without fundus photography [32]. Some investigators have argued that even the extreme phenotypes used by many groups are not sufficiently stringent, and that different criteria for controls could explain the failure of other investigators to replicate genetic association in the *TLR3* locus [33,34]. However, the high prevalence of a few hard drusen in aged individuals using both clinical examination and fundus photography suggests that more stringent criteria may not be possible. For example, 96% of all subjects (age 43 and older) had at least one drusen at the baseline in the Beaver Dam Eye Study [35].

A gold standard for defining a control could be based on clinical criteria (e.g., rate of progression to advanced disease) or some combination of biomarkers contributing to disease risk. Although genetic variation accounting for approximately 80% of the risk for AMD has now been identified, this information was not available at the time that the AREDS AMD severity scales were developed [36,37]. For the AREDS genome-wide scan data set, photographic grading was performed at a reading center using standardized approaches, and longitudinal follow-up detailing rates of progression allowed us to directly ask if “questionable control” subjects were more similar to cases or to controls. Figure 1 shows that the questionable control subjects were indistinguishable from the controls, based on risk allele frequencies at known AMD loci. Further, the rate of progression to advanced AMD for questionable control subjects was extremely low [27]. Thus, by both genetic and clinical criteria, questionable control subjects appear to be similar to controls. Our observation would be most valid in older subjects (≥60 years), and probably would not apply to younger individuals with a few hard drusen that are known to be at high risk for incident AMD [38]. Note that this observation will be useful to a variety of researchers studying the extensive clinical and epidemiologic data sets from the AREDS clinical trial.

We evaluated the AREDS GWAS for problems that could lead to faulty conclusions in genetic association studies. Genetic heterogeneity can inflate the significance of the association between SNPs and disease (type I errors) [28,39-41]. It is thus standard practice to perform genetic association analyses on homogeneous racial populations defined by self-reported race. Racial classifications defined using genetic data may be even more accurate than self-reported race. For example, we observed individuals that did not fit genetically with the self-reported race (Figure 2). Further, we observed residual substructure within the white population that artificially inflated the overall association with AMD in the AREDS GWAS (Figure 2 and Figure 3). Principal components methods statistically corrected for the substructure, reducing the genomic inflation to an acceptable level (Figure 3). Our analyses suggest that investigators using genetic data from the AREDS trial should account for population substructure in their studies.

Another problem in GWAS studies is the observation that differences in heterozygote frequencies between cases and controls can lead to type I errors, or false positive associations [26]. Thus, we performed here an analysis using a log-additive genetic model that is less sensitive to genotype distributions that differ primarily by heterozygote frequencies, and that is more sensitive to additive changes that are often used for analyses of complex traits. However, we acknowledge the limitation of the log-additive genetic model, which can be less powerful if the true model is not additive. Another problem can arise from relying on statistical tests of allele frequency differences between subject groups. While genotype tests are independent of the Hardy–Weinberg equilibrium (HWE), allele tests require the calculation of allele frequencies assuming HWE [42]. Deviation from HWE can arise from inaccurate genotyping or copy-number variation. Thus, we favor statistical models of inheritance based on genotype frequencies.

We observed statistically independent effects of two SNPs (rs4565845 and rs2014307) across the *ARMS2* locus at chromosome 10q26 and of two SNPs (rs433594 and rs2230199) across the *C3* locus. The independent effect of rs4565845 that we observed in the *PLEKHA1* gene adjacent to the *ARMS2* locus does not seem consistent with a well defined genomic region (e.g., haplotype). Others also have reported independent association with AMD in the *PLEKHA1* region, but it has not yet been possible to convincingly demonstrate an independent biologic effect, given the modest association with AMD, compared to the strong association in the haplotype block spanning the hypothetical gene *LOC387715* and the promoter of *HTRA1* [43]. It is hoped that these questions will be resolved in the future, as the biology of the *ARMS2* locus becomes better understood.

On the other hand, the independent effect of rs433594 in the *C3* gene appears to arise due to the strong association between AMD and a haplotype spanning exon 19 of *C3*. The genetic risk in the exon 19 region appears to be independent of the established genetic risks of non-synonymous SNPs in exons 3 and 9 of *C3*. Thus, it is possible that the genomic region spanning exon 19 contains a variant on a specific haplotype (see Independent genetic effects within the *ARMS2* and *C3* loci in Results section) that increases the risk of AMD. Re-sequencing studies are underway that may help address this question.

We identified 29 SNPs with p values greater than 0.0001 either before or after correction for known loci associated with AMD (Table 2). We attempted replication by genotyping each of these SNPs or a proxy (a nearby SNP in high linkage disequilibrium) in a larger, independent group of controls without AMD and in cases with AMD of similar ethnic background. However, none of the SNPs were definitely replicated in the independent group of subjects. Larger studies are ongoing and will be helpful in determining if the modest effects we observed upon replication (Table 2) and in our prior work with the AREDS GWAS may make a minor contribution to AMD [26]. Future work is needed to validate these results and to determine the functional relevance of these SNPs, if any.

Smoking is well established as a risk factor for both incident early AMD and advanced AMD [44-47]. In the AREDS GWAS, smoking was associated with both forms of advanced AMD only, presumably due to the stronger effect of smoking on the development of geographic atrophy and exudation. There have been inconsistent reports that the variants in the *ARMS2* locus may mediate [48-50] or interact [51,52] with the effect of smoking, but we did not observe an interaction between smoking and genetic risks in the AREDS subjects. Smoking appears to be an independent risk factor contributing to the development of AMD.

In summary, we reported the value of correcting for population stratification when working with the AREDS GWAS and have provided evidence supporting the use of questionable control subjects as controls in the AREDS GWAS study. Further, we presented further evidence that the contribution of smoking to the development of AMD is independent of genetic risks. Finally, we observed that the AREDS data set has evidence suggestive of a previously unknown genetic risk near exon 19 of the *C3* gene. Additional studies will be needed to determine if this observation can be replicated. There continues to be evidence for independent risks in the *ARMS2* locus.
